# 17-DMAG-Loaded HER2-Targeted Extracellular Vesicles Induce PARP/Caspase3-Mediated Apoptosis in Gastric Carcinoma

**DOI:** 10.3390/ijms27125377

**Published:** 2026-06-15

**Authors:** Sin Hye Park, Deok Yong Sim, Do Sang Lee, Chan Mi Lee, Joo Won Moon, Ji Won Choi, Dong Jin Kim

**Affiliations:** 1 Department of Gastrointestinal Surgery, Eunpyeong St. Mary’s Hospital, College of Medicine, The Catholic University of Korea, Seoul 03312, Republic of Korea; jwsh65@gmail.com; 2 Central Institute of Surgery, Seoul St. Mary’s Hospital, College of Medicine, The Catholic University of Korea, Seoul 06591, Republic of Korea; simdy0821@naver.com (D.Y.S.); dosangs@catholic.ac.kr (D.S.L.); cksal7873@naver.com (C.M.L.); m01410141@naver.com (J.W.M.); qrt97@naver.com (J.W.C.)

**Keywords:** gastric cancer, exosomes, HER2, 17-DMAG, apoptosis

## Abstract

Gastric cancer remains a major clinical challenge, underscoring the need for more effective drug delivery strategies. Approximately 10–20% of gastric cancers overexpress HER2, conferring aggressive tumor characteristics and poor survival, yet resistance to trastuzumab-based targeted therapy and limited intratumoral antibody penetration continue to restrict clinical outcomes. This study evaluated HER2-targeted exosomes as a delivery platform. Exosomes were engineered to express the p51 peptide, a high-affinity HER2-binding ligand, and loaded with 17-dimethylaminoethylamino-17-demethoxygeldanamycin (17-DMAG), a potent HSP90 inhibitor. The cellular uptake and antitumor efficacy of p51-Exo^17-DMAG^ were assessed in vitro using NCI-N87 and AGS cells and in vivo using a mouse xenograft model. p51-modified exosomes exhibited superior HER2 specific uptake. Treatment with p51-Exo^17-DMAG^ significantly increased apoptosis, as demonstrated by elevated PARP and caspase3 cleavage, and downregulated oncogenic signaling molecules, including p-AKT, CDK2, VEGF, and c-Myc. Furthermore, p51-Exo^17-DMAG^ increased the number of TUNEL-positive cells. In the NCI-N87 xenograft model, systemic administration of p51-Exo^17-DMAG^ significantly inhibited tumor growth without toxicity or histological damage to major organs. Tumor analysis confirmed increased apoptosis and reduced proliferation in vivo. These findings demonstrate that p51-engineered exosomes provide an efficient, selective, and safe platform for HER2-targeted delivery of 17-DMAG, offering a promising precision medicine strategy for HER2-positive gastric cancer.

## 1. Introduction

Gastric carcinoma is the fifth most common malignant cancer and the fifth leading cause of cancer-related mortality worldwide [1]. Current treatment strategies include surgery, chemotherapy, immunotherapy, and targeted therapy [2,3]. For patients with unresectable advanced or metastatic gastric cancer, fluoropyrimidine- and platinum-based doublet chemotherapy has long been the standard first-line backbone; however, it yields a median overall survival of less than one year, highlighting the limited efficacy of cytotoxic regimens alone [4,5]. Recent phase 3 trials have demonstrated that the addition of PD-1 inhibitors to standard chemotherapy significantly improves progression-free and overall survival in patients with HER2-negative advanced gastric cancer [5,6]. Nevertheless, durable responses remain limited, in part due to the heterogeneous and immunosuppressive tumor microenvironment that characterizes this disease [7]. Therefore, the development of novel therapeutic approaches that enhance antitumor efficacy while minimizing systemic toxicity remains a critical need.

Human epidermal growth factor receptor 2 (HER2) is an important biomarker and therapeutic target in gastric cancer [8]. Approximately 10–20% of patients with gastric cancer exhibit HER2 overexpression, which is often associated with more aggressive tumor behavior including higher rates of lymph node metastasis and distant metastasis, which contribute to poor overall prognosis [9,10]. The ToGA trial established trastuzumab, a monoclonal antibody targeting HER2, as the first-line targeted therapy for patients with HER2-positive advanced gastric cancer [11]. Despite the clinical success of trastuzumab, several patients eventually develop resistance to the treatment [12,13], and the limited penetration of large monoclonal antibodies into tumor tissues remains a major limitation [14,15]. These challenges underscore the need for advanced delivery systems that can bypass or overcome resistance mechanisms.

Exosomes are one of the smallest natural extracellular vesicles (30–150 nm) [16]. They are secreted by most cell types and play critical roles in intercellular communication by transferring proteins, lipids, and nucleic acids between cells [17]. Due to high biocompatibility, low immunogenicity, and intrinsic cargo-protective properties of the exosomes, they have emerged as promising next-generation drug delivery vehicles [18,19,20]. Surface modification or ligand engineering of exosomes enables tumor-specific targeting and enhances cellular uptake, improving their therapeutic efficacy [21]. To enhance their therapeutic potential in HER2-positive tumors, we engineered exosomes to express the p51 peptide (CDTFPYLGWWNPNEYRY), a high-affinity HER2-binding ligand [22]. This modification enables selective delivery of therapeutic cargo to HER2-overexpressing gastric cancer cells while minimizing off-target effects.

To exploit this platform, 17-dimethylaminoethylamino-17-demethoxygeldanamycin (17-DMAG), a potent inhibitor of heat shock protein 90 (HSP90), was selected as a therapeutic agent. HSP90 stabilizes multiple oncogenic client proteins [23], and its inhibition induces apoptosis [24], cell cycle arrest [25], and the suppression of migration and invasion [26], thereby inhibiting gastric cancer progression [27]. Also, 17-DMAG was selected because it is a potent, water-soluble HSP90 inhibitor known to target key oncogenic pathways in various cancers [28]. To our knowledge, no prior study has investigated the antitumor mechanisms of 17-DMAG delivered via HER2-targeted exosomes. Therefore, this study investigated whether p51-engineered exosomes loaded with 17-DMAG enhance targeted delivery, promote apoptosis, and suppress tumor progression in HER2-positive gastric cancer models.

## 2. Results

### 2.1. Characterization of HER2-Targeted Exosomes

**Figure 1:**
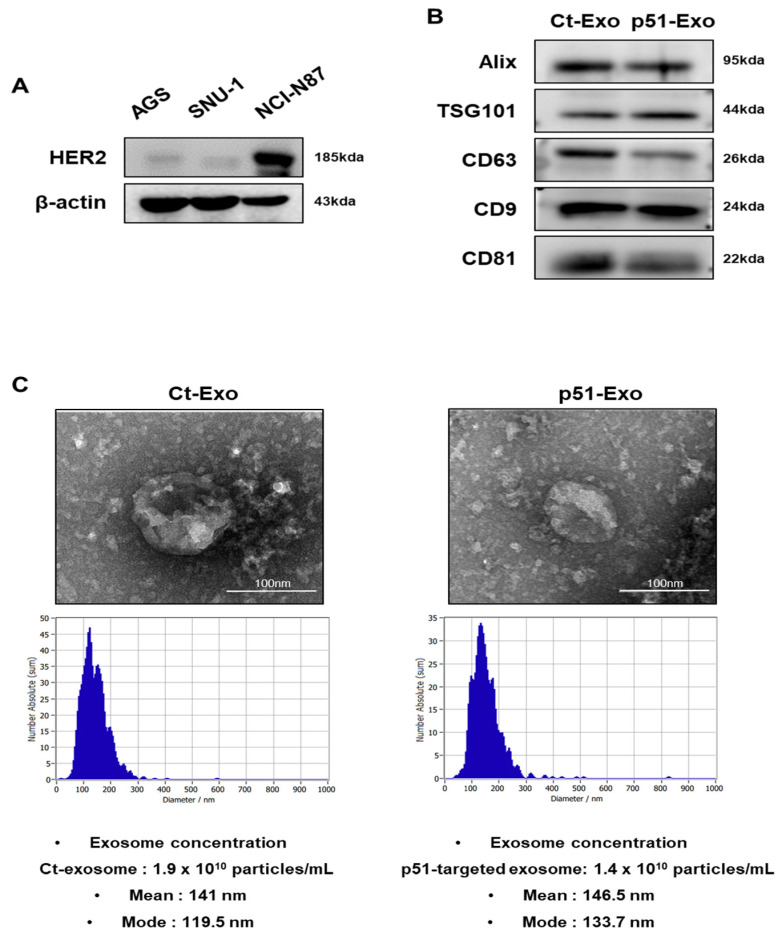
Characterization of HER2-targeted exosomes. (**A**) HER2 expression in AGS, SNU-1, and NCI-N87 gastric cancer cell lines. (**B**) Western blot analysis of exosomal markers Alix, TSG101, CD63, CD9, and CD81 in control exosomes (Ct-Exo) and HER2-targeted exosomes (p51-Exo). (**C**) Size distribution of nanoparticles determined by ZetaView analysis and transmission electron microscopy (TEM) images of Ct-Exo and p51-Exo, revealing round-to-ovoid vesicles with diameters of approximately 100–200 nm. Scale bar = 100 nm.

HER2-targeted exosomes were generated by engineering HEK-293T cells to express the HER2-binding p51 peptide. Basal HER2 expression was evaluated in AGS, SNU-1, and NCI-N87 gastric cancer cell lines. Among these, NCI-N87 cells exhibited the highest HER2 expression (Figure 1A). Therefore, NCI-N87 cells were selected for subsequent experiments. The expression of canonical exosome markers (Alix, TSG101, CD63, CD9, and CD81) was analyzed by Western blot analysis. The expression levels of these markers were compared in control exosomes (Ct-Exo) and HER2-targeted exosomes (p51-Exo), indicating that p51 modification did not alter exosomal marker expression (Figure 1B). Transmission electron microscopy (TEM) revealed similar nanoscale vesicular morphology in both groups (Figure 1C). Nanoparticle tracking analysis showed a mean diameter of 141 nm and a concentration of 1.9 × 10^10^ particles/mL for Ct-Exo, and 146.5 nm with a concentration of 1.4 × 10^10^ particles/mL for p51-Exo. These findings confirm the successful generation and isolation of HER2-targeted exosomes.

### 2.2. Cellular Uptake of HER2-Targeted Exosomes in Gastric Cancer Cells

**Figure 2:**
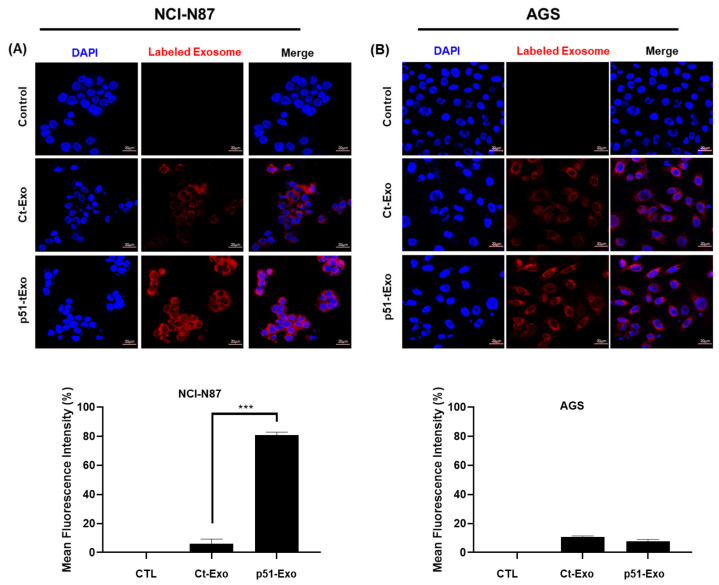
Cellular uptake of HER2-targeted exosomes in gastric cancer cells. (**A**,**B**) Uptake of DiL-labeled Ct-Exo and p51-Exo in NCI-N87 and AGS cells assessed by fluorescence microscopy. Red fluorescence indicates DiL-labeled exosomes, and blue fluorescence indicates DAPI stained nuclei. Quantitative analysis of the mean fluorescence intensity (MFI) of internalized exosomes per cell. Scale bar = 20 μm. *** *p* < 0.001 versus untreated control.

The cellular uptake of exosomes was evaluated using 1,1′-dioctadecyl-3,3,3′,3′-tetramethylindocarbocyanine perchlorate (DiI)-labeled Ct-Exo and p51-Exo in gastric cancer cells. In AGS cells with low HER2 expression, no significant difference in DiI fluorescence was observed between Ct-Exo and p51-Exo. In contrast, NCI-N87 cells exhibited markedly increased DiI fluorescence following treatment with p51-Exo compared with Ct-Exo, indicating enhanced uptake of targeted exosomes (Figure 2A,B). These findings demonstrate preferential accumulation of HER2-targeted exosomes in HER2-overexpressing gastric cancer cells, supporting effective HER2-specific targeting.

### 2.3. p51-Exo^17-DMAG^ Induced Apoptosis and Suppressed Proliferation in NCI-N87 Cells

**Figure 3:**
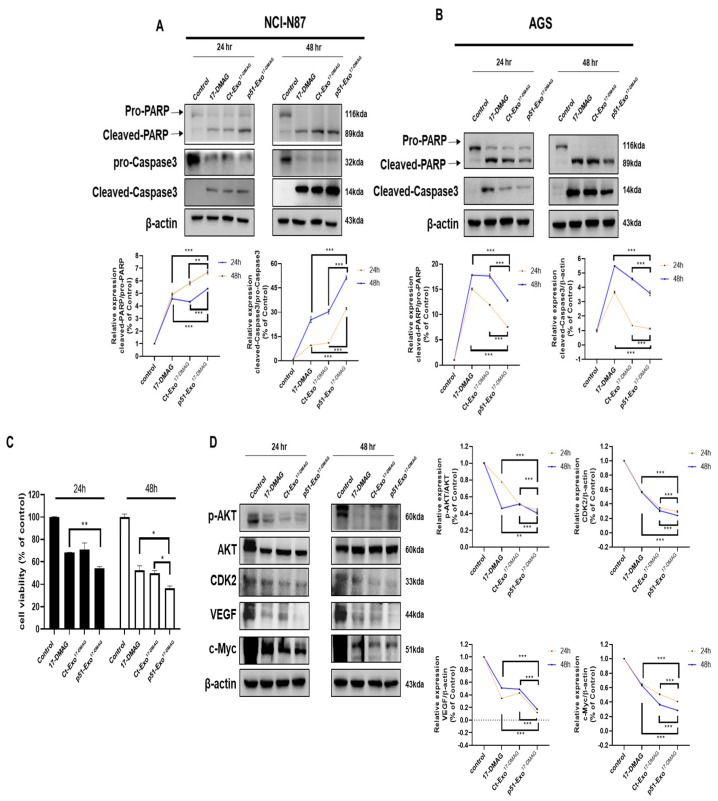
Effects of 17-DMAG-loaded p51-targeted exosomes on apoptosis and proliferation in NCI-N87 cells. (**A**,**B**) Expression of PARP, pro-caspase3, and cleaved-Caspase3 in NCI-N87 and AGS cells. (**C**) Cytotoxic effects of 17-DMAG, Ct-Exo^17-DMAG^, and p51-Exo^17-DMAG^ in NCI-N87 cells assessed by CCK-8 assay following 24 and 48 h of treatment. * *p* < 0.05, ** *p* < 0.01, *** *p* < 0.001 versus untreated control. (**D**) Expression of p-AKT, CDK2, VEGF, and c-Myc. Cells were treated with 17-DMAG, Ct-Exo^17-DMAG^, and p51-Exo^17-DMAG^ for 24 and 48 h and analyzed by Western blot analysis. Protein levels were normalized to β-actin, and experiments were performed in triplicate.

Ct-Exo and p51-Exo loaded with 100 nM 17-DMAG were designated as Ct-Exo^17-DMAG^ and p51-Exo^17-DMAG^, respectively; this specific concentration was chosen based on the optimal cytotoxic and apoptotic efficacy observed in Supplementary Figure S1. Western blot analysis was performed following treatment with 17-DMAG, Ct-Exo^17-DMAG^, and p51-Exo^17-DMAG^ for 24 and 48 h in NCI-N87 and AGS gastric cancer cells. Treatment with p51-Exo^17-DMAG^ significantly increased cleaved-PARP and cleaved-caspase3 levels and reduced pro-PARP and pro-caspase3 in a time-dependent manner compared with other groups in HER2-overexpressing NCI-N87 cells (all *p* < 0.05; Figure 3A). Conversely, in AGS cells with low HER2 expression, p51-Exo^17−DMAG^ did not exhibit enhanced apoptotic effects compared to free 17-DMAG or non-targeted Ct-Exo^17−^DMAG (Figure 3B). These results indicate that the therapeutic enhancement of p51-Exo^17−^DMAG is strictly dependent on HER2 expression, confirming the target-specific cell death induced by our engineered exosome platform.

To evaluate the impact of p51-Exo17-DMAG on key oncogenic signaling pathways downstream of HER2, we examined the expression of p-AKT, CDK2, VEGF, and c-Myc markers representing the major survival, proliferative, and angiogenic axes activated by HER2 overexpression in gastric cancer. p51-Exo^17-DMAG^ markedly reduced cell viability in a time-dependent manner in NCI-N87 cells (Figure 3C). Furthermore, p51-Exo^17-DMAG^ markedly reduced the expression of survival- and proliferation-associated proteins, including phosphorylated protein kinase B (p-AKT), cyclin-dependent kinase 2 (CDK2), vascular endothelial growth factor (VEGF), and c-Myc (all *p* < 0.05; Figure 3D). These results indicate that HER2-targeted delivery of 17-DMAG enhances apoptotic signaling and suppresses oncogenic pathways in NCI-N87 cells.

### 2.4. p51-Exo^17-DMAG^ Significantly Enhanced the Number of TUNEL Green-Fluorescent Cells in NCI-N87 Cells

**Figure 4:**
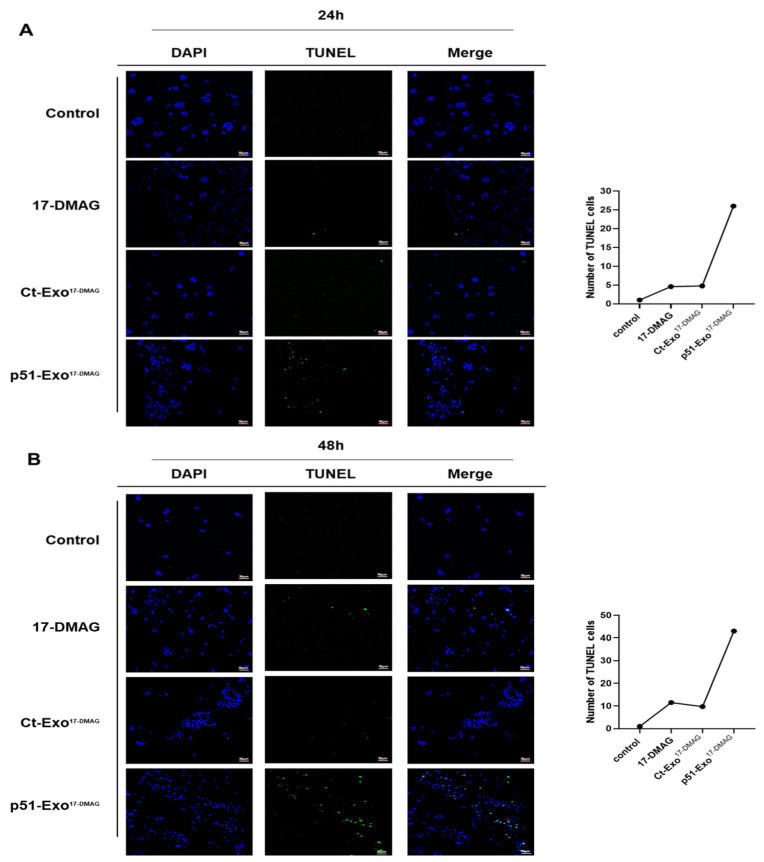
Effect of 17-DMAG-loaded p51-targeted exosomes on the number of TUNEL-positive cells in NCI-N87 cells. NCI-N87 cells treated with 17-DMAG, Ct-Exo^17-DMAG^, and p51-Exo^17-DMAG^ for 24 (**A**) and 48 h (**B**) were subjected to TUNEL staining and then were observed using an Olympus FLUOVIEW FV10i Confocal microscope. Scale bar = 20 μm.

To examine whether the cytotoxic effect of *p51-Exo^17-DMAG^* is exerted by apoptosis, TUNEL assay was conducted in NCI-N87 cells Here, *p51-Exo^17-DMAG^* significantly enhanced the number of TUNEL green-fluorescent cells compared to untreated control for 24 and 48 h in NCI-N87 cells (Figure 4A,B).

### 2.5. p51-Exo^17-DMAG^ Suppressed the Growth of NCI-N87 Cells in a Xenograft Tumor Model

**Figure 5:**
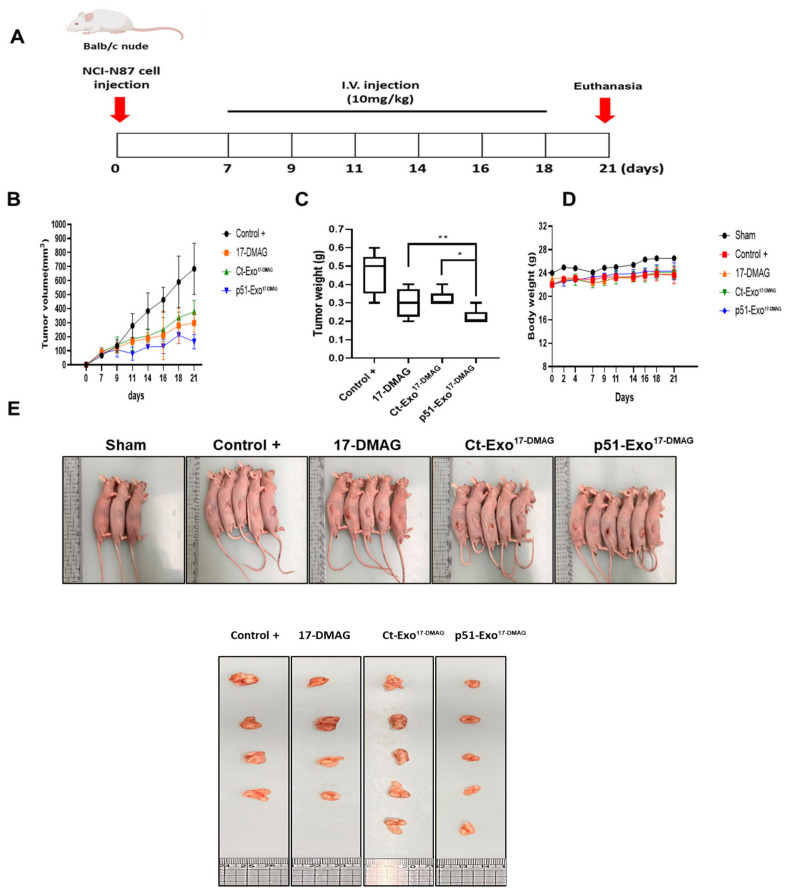
17-DMAG-loaded p51-targeted exosomes suppress tumor growth in an NCI-N87 xenograft model. (**A**) Schematic of the animal study design. The schematic diagram was created with BioRender.com. (**B**) Body weight changes. (**C**) Tumor volume. (**D**) Tumor weight. Groups include sham (*n* = 3), control (*n* = 5), 17-DMAG (*n* = 5), Ct-Exo^17-DMAG^ (*n* = 5), and p51-Exo^17-DMAG^ (*n* = 6). * *p* < 0.05, ** *p* < 0.01 versus untreated control. (**E**) Representative images of tumor-bearing BALB/c athymic nude mice.

An NCI-N87 xenograft model was established in BALB/c male athymic nude mice (Figure 5A). Tumor-bearing mice received intravenous (IV) tail vein administration of 17-DMAG, Ct-Exo^17-DMAG^, and p51-Exo^17-DMAG^. Treatment with p51-Exo^17-DMAG^ significantly suppressed tumor growth compared with control, 17-DMAG (*p* < 0.01), and Ct-Exo^17-DMAG^ groups (*p* < 0.05; Figure 5B,E). Consistently, tumor volume was also significantly reduced in the p51-Exo^17-DMAG^ group (Figure 5C). Importantly, no significant changes in body weight were observed, indicating minimal systemic toxicity (Figure 5D). Histological analysis of major organs showed no pathological alterations in liver or spleen tissues (Supplementary Figure S2). These findings demonstrate effective tumor suppression by p51-Exo^17-DMAG^ without detectable toxicity.

### 2.6. p51-Exo^17-DMAG^ Increased Apoptosis in Xenograft Tumor Model

**Figure 6:**
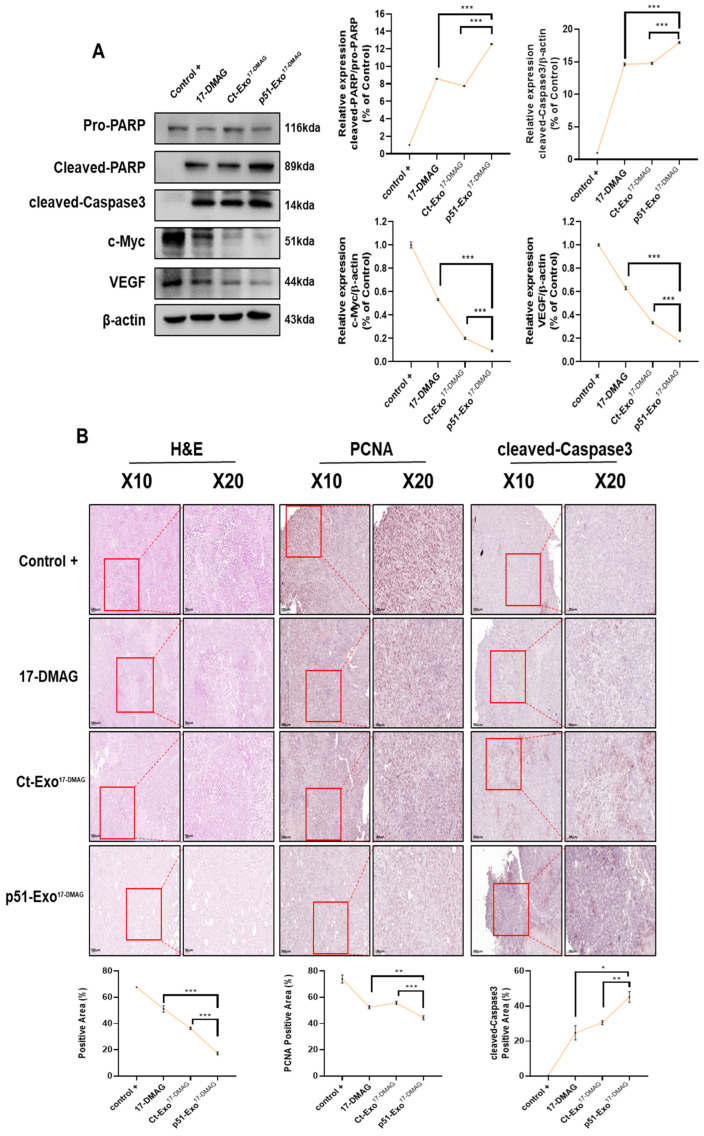
Effects of 17-DMAG-loaded p51-targeted exosomes on apoptosis in NCI-N87 xenograft tumors. (**A**) Western blot analysis of tumor lysates from control, 17-DMAG, Ct-Exo^17-DMAG^, and p51-Exo^17-DMAG^ groups showing expression of PARP, cleaved-caspase3, c-Myc and VEGF. *** *p* < 0.001 versus untreated control. (**B**) Representative hematoxylin and eosin staining and immunohistochemistry for PCNA and cleaved-caspase3 in xenograft tumor tissues. * *p* < 0.05, ** *p* < 0.01, and *** *p* < 0.001 versus untreated control. Scale bars = 50 and 100 μm.

Tumor tissues from xenograft models were analyzed to assess apoptotic signaling following treatment. Western blot analysis showed reduced expression of pro-PARP, c-Myc, and VEGF, along with increased cleaved-PARP and cleaved-caspase3 in the p51-Exo^17-DMAG^ group compared with other groups (all *p* < 0.05; Figure 6A). Immunohistochemical analysis demonstrated decreased proliferating cell nuclear antigen (PCNA) expression and increased cleaved-caspase3 staining in tumors treated with p51-Exo^17-DMAG^, compared to other groups (Figure 6B). These findings indicate that HER2-targeted delivery of 17-DMAG increased apoptosis and suppressed tumor proliferation in vivo.

## 3. Discussion

Gastric cancer remains a leading cause of cancer-related mortality worldwide, with limited therapeutic options and poor prognosis for patients with advanced or metastatic disease. Although advances in cytotoxic chemotherapy and targeted therapy, including trastuzumab, have improved survival in selected patients, drug resistance and inefficient tumor delivery continue to limit complete responses. Therefore, the development of targeted delivery systems capable of selective intracellular drug delivery while minimizing systemic toxicity in HER2-overexpressing tumors remains essential.

Selective targeting of HER2-positive tumor cells is critical for improving therapeutic efficacy. The p51 peptide, which exhibits high affinity and specificity for the extracellular domain of HER2, was utilized for exosome surface engineering [22]. The results of the present study demonstrated that p51-modified exosomes were preferentially internalized by HER2-overexpressing NCI-N87 cells compared with HER2-low AGS cells, indicating enhanced targeting efficiency. These findings suggest that p51-modified exosomes could provide a more efficient route for intracellular drug delivery than conventional chemotherapy or monoclonal antibodies. Although the ToGA trial established trastuzumab-based therapy as the standard of care for HER2-positive gastric cancer [8], resistance development and limited tumor penetration of large antibodies remain significant clinical obstacles. Furthermore, despite the survival benefit conferred by PD-1 inhibitor-based combination regimens in HER2-negative disease [5,6], a substantial proportion of patients fail to achieve durable responses, reflecting the immunosuppressive and heterogeneous tumor microenvironment [7]. In this context, exosome-based targeted delivery represents a complementary strategy to enhance intratumoral drug concentration.

Exosomes are promising nanocarriers due to intrinsic biocompatibility, low immunogenicity, membrane fusion capability, and protection of therapeutic cargo from degradation [20,21,29]. Surface engineering strategies, including peptide modification, enhance tumor-selective uptake and delivery efficiency in various cancers [30,31]. In addition, peptide-functionalized exosomes, such as those incorporating p51, may improve pharmacokinetics by promoting tumor accumulation while reducing systemic exposure [32]. In the context of the tumor microenvironment, the small size of exosomes further improves tumor penetration, which is a limiting factor for large monoclonal antibodies, such as trastuzumab.

Previous studies have demonstrated that 17-DMAG induces apoptosis through HSP90 inhibition, leading to destabilization and proteasomal degradation of oncogenic client proteins in gastric cancer cells [33,34,35]. Consistent with these findings, 17-DMAG treatment increased cleaved-PARP and cleaved-caspase3 levels in gastric cancer cells (Supplementary Figure S1) and decreased p-AKT, CDK2, VEGF, and c-Myc expression in HER2-overexpressing NCI-N87 cells. Notably, HER2-targeted exosomes more effectively upregulated apoptosis-related proteins and suppressed proliferation-associated proteins, including p-AKT, CDK2, VEGF, and c-Myc, compared with 17-DMAG alone. To further clarify the molecular mechanism underlying this enhanced therapeutic efficacy, we investigated the alterations in these key downstream effectors of the HER2 signaling pathway. Persistent activation of HER2 triggers the PI3K/AKT cascade [36,37], where p-AKT serves as a central hub promoting cell survival and metabolic reprogramming [38]. Downstream of this axis, c-Myc, a potent oncogenic transcription factor [39,40,41], is stabilized to accelerate tumor growth and drive the cell cycle transition through the activation of CDK2 [42,43]. Furthermore, aberrant HER2 signaling is well-known to upregulate VEGF [44,45], a master regulator of tumor angiogenesis that facilitates hypervascularization and metastasis [46,47,48,49].

Therefore, the simultaneous downregulation of p-AKT, CDK2, VEGF, and c-Myc following p51-exosome treatment provides strong molecular evidence that our extracellular vesicle-based platform successfully blocks the HER2 receptor, leading to a multi-pronged suppression of cancer cell survival, cell cycle progression, and angiogenic potential.

These findings suggest that targeted exosome delivery enhances local drug concentration within tumors, thereby maximizing the modulation of multiple signaling pathways.

Furthermore, the in vivo results of this study highlight a strong clinical potential of the exosomes. Significant inhibition of tumor growth in the xenograft model, accompanied by stable body weights and the absence of histological toxicity in major organs including the liver and spleen (Supplementary Figure S2), supports the safety and efficacy of p51-Exo^17-DMAG^. Although 17-DMAG has demonstrated promising outcomes in clinical studies, its application has been limited by dose-limiting toxicities [50]. These data suggest that exosome-based delivery may broaden the therapeutic window by enhancing tumor-specific drug accumulation while minimizing off-target effects.

Consistent with these findings, tumor tissue analysis showed increased cleaved-caspase3 expression and reduced PCNA levels following p51-Exo^17-DMAG^ treatment, demonstrating effective in vivo delivery. These results demonstrate that the p51-Exo^17-DMAG^ enhances proapoptotic and antiproliferative effects in vivo. Collectively, the HER2-targeted exosome platform shows promise as a therapeutic agent that enhances treatment precision and efficacy in HER2-overexpressing gastric cancer.

There are several limitations in this study. First, although 17-DMAG loading was achieved by passive incubation, quantitative assessment of encapsulation efficiency and comparison with alternative loading methods (e.g., electroporation or sonication) are required to optimize the delivery system. Second, the release profile of 17-DMAG from the exosomal carrier under physiological conditions remains unclear, and detailed characterization of release kinetics is necessary to inform dosing strategies and predict sustained therapeutic effects. Third, validation in patient-derived xenograft models is warranted to further elucidate the tumor heterogeneity and microenvironment of human gastric cancer. Fourth, the use of athymic nude mice limits the evaluation of the potential immunogenicity of engineered exosomes and their interaction with a functional immune system. Therefore, comprehensive evaluations in immunocompetent models are necessary to assess the long-term safety, half-life, and potential pro-inflammatory effects of these engineered vesicles before clinical translation. Fifth, the mechanistic characterization of apoptosis in this study was focused on cleaved PARP and cleaved caspase-3 as executioner markers. The potential contribution of upstream mitochondrial pathway effectors including ROS generation, mitochondrial membrane potential changes, and Bcl-2 family protein regulation was not directly assessed. Prior studies have demonstrated that anticancer agents induce apoptosis in gastrointestinal cancer cells through these mitochondrial mechanisms [51,52,53,54], suggesting that similar pathways may contribute to the antitumor activity of p51-Exo17-DMAG. Future studies incorporating these measurements would provide a more comprehensive understanding of the underlying cell death mechanisms.

Overall, HER2-targeted exosomes represent an effective platform for the selective delivery of small-molecule anticancer agents. Although 17-DMAG was used as a model cargo in this study, the findings support the broader clinical potential of engineered exosomes in precision oncology.

## 4. Materials and Methods

### 4.1. Cell Culture

Human gastric carcinoma cell lines NCI-N87 (No. 60113) and AGS (No. 21739), obtained from Korean Cell Line Bank (Seoul, Republic of Korea), were cultured in RPMI-1640 supplemented with 10% fetal bovine serum (FBS) and 1% penicillin in a humidified incubator at 37 °C with 5% CO_2_.

### 4.2. Preparation of Exosomes

HEK-293T cells were used for the production of HER2-targeted exosomes. Cells were seeded at a density of 3 × 10^6^ cells in 100 mm culture dishes. After 24 h of incubation, the pDisplay-p51 vector encoding the HER2-binding peptide p51 (CDTFPYLGWWNPNEYRY) was transfected into the cells using Lipofectamine 3000 (Invitrogen, Carlsbad, CA, USA) according to the manufacturer’s protocol. Briefly, 4 μg of plasmid DNA was mixed with transfection reagent at a 1:2 (μg:μL) ratio and incubated for 15 min at room temperature before adding to the cells. After 6 h, the medium was replaced with fresh DMEM containing 10% FBS. Cells were cultured for an additional 24 and 48 h to allow exosome production. Conditioned medium was then collected and centrifuged to remove cells and debris, followed by ultracentrifugation at 120,000× *g* for 120 min at 4 °C using a Beckman Coulter Optima ultracentrifuge. Exosome pellets were washed once with phosphate-buffered saline (PBS), resuspended in PBS, and stored at −80 °C until further use. For drug loading, purified exosomes were incubated with 17-DMAG at 36 °C for 2 h.

### 4.3. Transmission Electron Microscopy

Control and p51-modified exosomes were fixed in 2% paraformaldehyde. Morphological characterization was performed using a Hitachi HT7800 transmission electron microscope (Hitachi, Tokyo, Japan) with support from a skilled technician at The Catholic University.

### 4.4. Nanoparticle Tracking Analysis

Extracellular vesicles derived from HEK-293T cells were diluted 1:10 in PBS and analyzed using a ZetaView instrument (Particle Metrix, Wildmoos, Germany). After stabilizing the flow rate, particle Brownian motion was recorded using the instrument’s automatic tracking mode. Measurements were performed in triplicate, and the mean particle size distribution and concentration were calculated.

### 4.5. Exosome Uptake Assays

NCI-N87 and AGS cells were seeded at a density of 5 × 10^5^ cells per well in 8-well chamber slides (SPL Life Sciences, Gyeonggi, Republic of Korea) and cultured overnight. Cells were treated with DiI-labeled exosomes (1 × 10^8^ particles/mL) for 24 h at 37 °C in 5% CO_2_. Following incubation, cells were washed twice with PBS and fixed with 4% paraformaldehyde for 10 min at room temperature. Nuclei were stained with DAPI-containing mounting medium (Vector Laboratories, Burlingame, CA, USA). The cellular uptake of DiI-labeled exosomes was visualized using a confocal laser scanning microscope (LSM700; Carl Zeiss, Oberkochen, Germany), and fluorescence intensity was qualitatively compared between groups.

### 4.6. Cell Viability Assay

The cytotoxicity of 17-DMAG and 17-DMAG-loaded exosomes was evaluated using a CCK-8 assay (DOJINDO Laboratories, Kumamoto, Japan) according to the manufacturer’s instructions.

For the drug treatment groups, NCI-N87, AGS, and SNU-1 gastric cancer cells (5  ×  10^3^ cells/well) were seeded in 96-well plates and treated with varying concentrations of 17-DMAG for 24 and 48 h. For the exosome treatment group, NCI-N87 cells were treated with different concentrations of exosomes for the specified time periods. Following incubation, the cells were exposed to the CCK-8 solution for 40 min. Absorbance was then measured at 450 nm using a BioTek Synergy HTX Multi-Mode Microplate Reader (Agilent, BioTek Instruments, Winooski, VT, USA). Cell viability was calculated and expressed as a percentage relative to the respective untreated controls.

### 4.7. Western Blot Analysis

NCI-N87 cells, exosomes, or xenograft tumor tissues were lysed in RIPA buffer (Thermo Scientific, Rockford, IL, USA) supplemented with protease inhibitor cocktail and phosphatase inhibitors (Roche, Mannheim, Germany). Protein concentrations were determined using a Pierce^TM^ BCA protein assay kit (Thermo Scientific). Equal amounts of protein samples were separated by 8–15% sodium dodecyl sulfate–polyacrylamide gel electrophoresis and transferred to nitrocellulose membranes. Membranes were blocked with tris-buffered saline, supplemented with 0.1% Tween 20 (TBST) diluted 5% skim milk, for 1 h at room temperature. Membranes were incubated overnight at 4 °C with primary antibodies against PARP (Cat. No. 9542, Cell Signaling Technology, Danvers, MA, USA), caspase3 (Cat. No. ab13585, Abcam, Waltham, MA, USA), HER2 (Cat. No. 2242, Cell Signaling Technology), Alix (Cat. No. 2171, Cell Signaling Technology), TSG101 (Cat. No. ab77671, Abcam), CD63 (Cat No sc-365604, Santa Cruz Biotechnology, Dallas, TX, USA), CD9 (Cat. No. 13403, Cell Signaling Technology), CD81 (Cat. No. 56039, Cell Signaling Technology), p-AKT (Cat. No. 9271, Cell Signaling Technology), CDK2 (Cat. No. ab77671, Abcam), VEGF (Cat No ab1316, Abcam), c-Myc (Cat No ab32072, Abcam), and β-actin (Cat No sc-47778, Santa Cruz Biotechnology, Dallas, TX, USA), diluted in 3% bovine serum albumin in TBST. Membranes were washed thrice with TBST (10 min each time) and incubated with HRP-conjugated secondary antibodies for 2 h at room temperature. Protein expression was visualized using Western BLoT Hyper HRP Substrate (Takara, Kusatsu, Japan).

### 4.8. TUNEL Assay

TUNEL was considered as a feature of apoptosis [55]. Cell death was assessed using the DeadEnd^TM^ Fluorometric TUNEL system kit according to Promega’s instructions (Cat No G3250, Promega, Madison, WI, USA). In brief, NCI-N87 cells treated with 17-DMAG, Ct-Exo^17-DMAG^, and p51-Exo^17-DMAG^ for 24 and 48h were exposed to 4% paraformaldehyde, permeabilization solution and TUNEL assay mixture. Finally, the TUNEL-stained cells were photographed using a FLUOVIEW FV10i confocal microscope (Olympus, Tokyo, Japan).

### 4.9. Xenograft Models and Immunohistochemistry

All animal experiments were approved by the Institutional Animal Care and Use Committee of the Catholic University of Korea (CMCIBC-2024-055-02). For the xenograft model, NCI-N87 cells were suspended in serum-free medium and mixed with Matrigel (Corning, Bedford, MA, USA) in a 1:1 ratio. The cell (1 × 10^7^ in l00 μL) suspension was injected subcutaneously into the right flank of athymic BALB/c nude mice. In vivo antitumor effects were evaluated across five groups: sham (*n* = 3), control (*n* = 5), 17-DMAG (*n* = 5), Ct-EXO^17-DMAG^ (*n* = 5), and p51-EXO^17-DMAG^ (*n* = 6). Treatments were administered via intravenous injections through the tail vein every 2 days for 3 weeks, with a total injection volume of 120 µL. Tumor growth was monitored for 21 days. All mice were euthanized 21 days after tumor implantation. Tumors were excised, photographed, and weighed. Histological analysis was performed using hematoxylin and eosin staining. Immunohistochemistry was conducted on tumor sections using antibodies against PCNA (Cat No. sc-376228, Santa Cruz Biotechnology) and cleaved- caspase3 (Cat no. sc-40, Santacruz Biotechnology).

### 4.10. Statistical Analysis

All data were expressed as means ± standard deviation. Statistical significance was evaluated using one-way analysis of variance with the GraphPad Prism 8 software. A *p*-value of <0.05 was considered statistically significant. All experiments were performed in triplicate and independently repeated thrice.

## 5. Conclusions

In conclusion, a novel HER2-targeted exosomal delivery system was developed to enhance the therapeutic efficacy and safety of 17-DMAG in gastric cancer. Engineering exosomes with the p51 peptide enabled selective targeting of HER2-overexpressing cancer cells, resulting in markedly increased intracellular drug uptake. This targeted approach enhanced PARP/caspase3-mediated apoptosis and suppressed oncogenic pathways, including p-AKT and c-Myc in vitro, and induced significant tumor growth inhibition in vivo without detectable systemic toxicity.

## Data Availability

The original contributions presented in this study are included in the article/Supplementary Material. Further inquiries can be directed to the corresponding author.

## Supplementary Materials

The following supporting information can be downloaded at: https://www.mdpi.com/article/10.3390/ijms27125377/s1.

## Abbreviations

The following abbreviations are used in this manuscript:

HER2	human epidermal growth factor receptor 2
17-DMAG	17-dimethylaminoethylamino-17-demethoxygeldanamycin
CCK-8	cell counting kit 8
TEM	transmission electron blot microscopy
NTA	nanoparticle tracking analysis
Dil	1,1′-dioctadecyl-3,3,3′,3′-tetramethylindocarbocyanine perchlorate
AKT	phosphorylated protein kinase B
CDK2	cyclin-dependent kinase 2
VEGF	vascular endothelial growth factor
IV	intravenous
PCNA	proliferating cell nuclear antigen
