# Supplementary material for: 17-DMAG-Loaded HER2-Targeted Extracellular Vesicles Induce PARP/Caspase3-Mediated Apoptosis in Gastric Carcinoma
Source: Int J Mol Sci. 2026 Jun 15;27(12):5377. doi: 10.3390/ijms27125377 (PMC13300701; doi:10.3390/ijms27125377)
Supplement: Supplementary file 1 [file ijms-27-05377-s001.zip › ijms-4306570-supplementary.pdf]

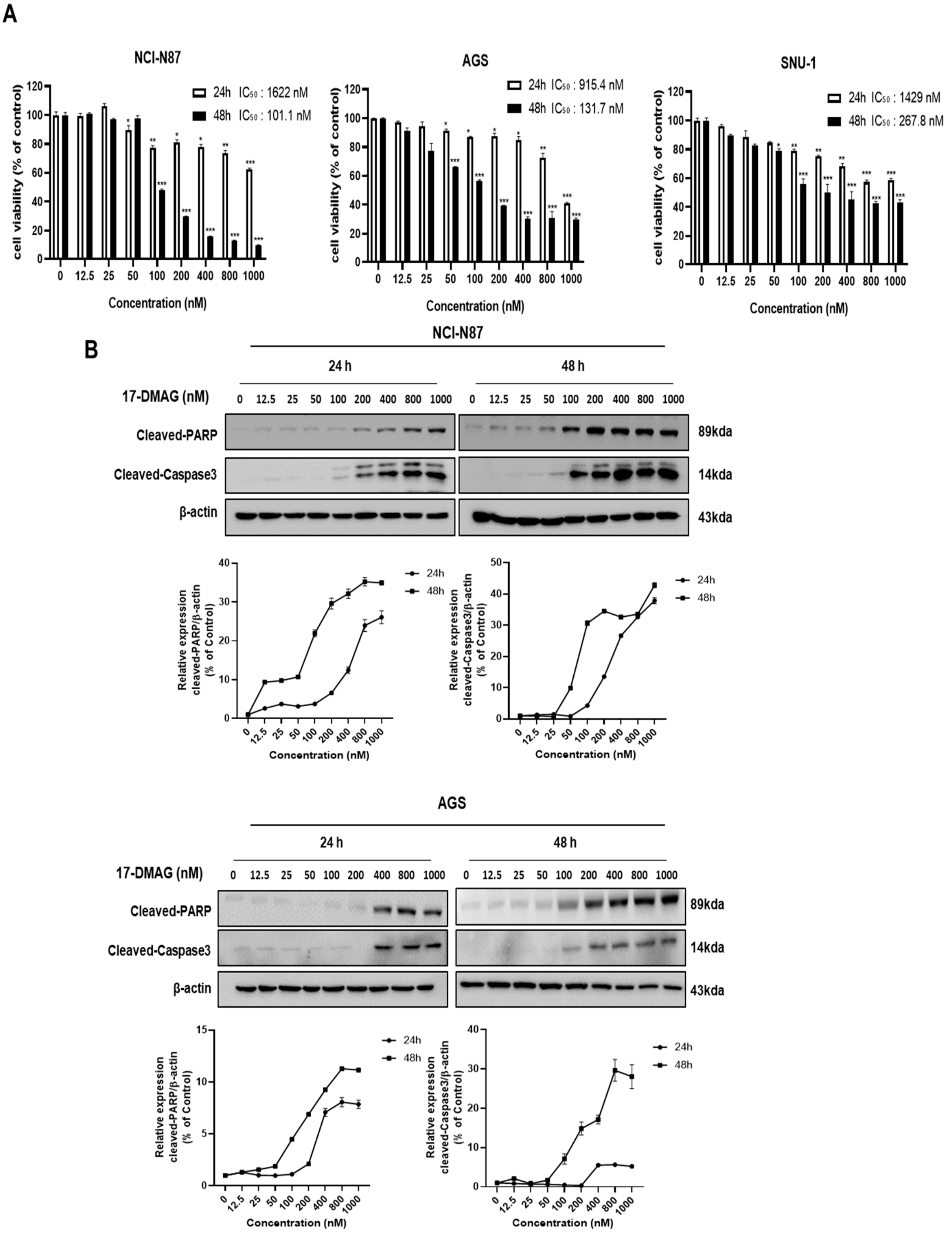

Figure S1. Effect of 17-DMAG on cytotoxicity and apoptosis in gastric cancer cells. (A) Cytotoxic effects of 17-DMAG in NCI-N87, AGS, and SNU-1 gastric cancer cells assessed by CCK-8 assay following 24 and 48 h of treatment. *$p < 0.05$, **$p < 0.01$, ***$p < 0.001$ versus untreated control (B) Expression of cleaved-PARP and cleaved- caspase3 in NCI-N87 and AGS cells following 17-DMAG for 24 and 48 h, analyzed by western blotting. Protein levels were normalized to β-actin. Data are presented as mean ± SD from three independent experiments.

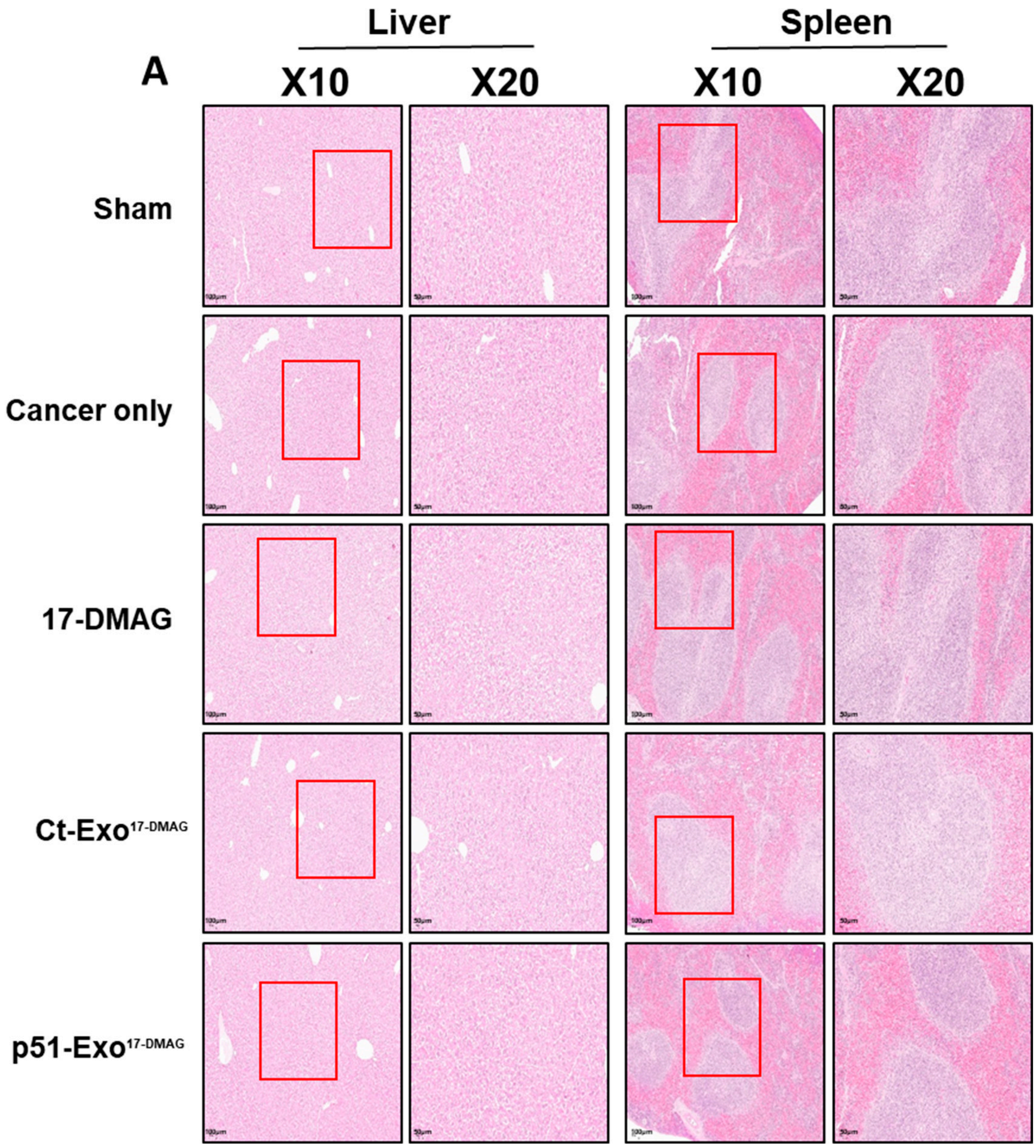

Figure S2. Hematoxylin and eosin staining in liver and spleen of tumor-bearing BALB/c athymic nude mice. Scale bars = 50 and 100 μm.
